# Liquid–Liquid Phase Separation Prediction of Proteins in Salt Solution by Deep Neural Network

**DOI:** 10.3390/biom13010042

**Published:** 2022-12-26

**Authors:** Suwen Wei, Yanwei Wang, Guangcan Yang

**Affiliations:** Department of Physics, Wenzhou University, Wenzhou 325035, China

**Keywords:** cloud point temperature, LLPS, BSA, lysozyme, machine learning

## Abstract

Liquid–liquid phase separation (LLPS) underlies the formation of membrane-free organelles in eukaryotic cells and plays an important role in the development of some diseases. The phase boundary of metastable liquid–liquid phase separation as well as the cloud point temperature of some globular proteins characterize the phase behavior of proteins and have been widely studied theoretically and experimentally. In the present study, we used a regression and classification neural network to deal with the phase behavior of lysozyme and bovine serum albumin (BSA). We predicted the cloud point temperature and solubility of a lysozyme solution containing sodium chloride by regression and the reentrant phase behavior of BSA in YCl_3_ solution containing a surfactant dodecyl dimethyl amine oxide (DDAO) by classification. Specifically, our network model is capable of predicting (a) the solubility of lysozyme in the range: pH 4.0–5.4, temperature 0–25 °C, and NaCl concentration 2–7% (*w*/*v*); (b) the cloud point temperature of lysozyme in the range: pH 4.0–4.8, NaCl concentration 2–7%, and lysozyme concentration 0–400 mg/mL; and (c) the phase behavior of BSA in the range: DDAO 1–60 mM, BSA 30–100 mg/mL, and YCl_3_ 1–20 mM. We experimentally tested the model at some prediction points with a high accuracy, which means that deep neural networks can be applicable in qualitative and quantitive analysis of liquid–liquid phase separation.

## 1. Introduction

Metastable liquid–liquid phase separation (LLPS) in a protein solution is a basic biophysical phenomenon [1], which provides a mechanism for the formation of membrane-less organelles and for the coordination of cytoskeletal regulatory molecules in living cells [2,3]. Dysregulated LLPS can also lead to abnormal protein aggregation, which is closely related to some diseases such as cancer and neurodegeneration [4,5].

Recent theoretical analysis and numerical simulations show that nucleation of crystals of some proteins—such as lysozyme, a typical globular protein—proceed in two steps under certain conditions: (a) the formation of a droplet of dense liquid and (b) nucleating a periodic crystal within the droplet [6]. When LLPS occurs, the protein solution separates into a dense, protein-rich phase and a dilute, protein-poor phase. It can be regulated by many factors such as adding a salt and adjusting the temperature of solution. The former phenomenon is called salting-out and it is the basis of the salt-induced precipitation and crystallization process. Salt-induced precipitation is related to the solubility of protein and depends on protein type as well as concentration, pH, and temperature [7]. One of the empirical parameters for quantifying protein–protein interaction is the cloud point temperature (CPT) of the protein. This temperature represents the point at which the protein solution shows LLPS. Through the cloud point temperature, we can obtain the liquid–liquid coexistence curve and the phase diagram. As the concentration of protein or salt changes, the solution changes from a homogeneous mixed state to a liquid–liquid phase separation state (where the salt concentration is defined as a low critical salt concentration) and then from a liquid–liquid phase separation state to a uniform mixed state (where the salt concentration is defined as a high critical salt concentration), implying that the reentrant condensation (RC) phase behavior has been confirmed between several proteins and the chloride of trivalent salts [8,9].

**Figure 1:**
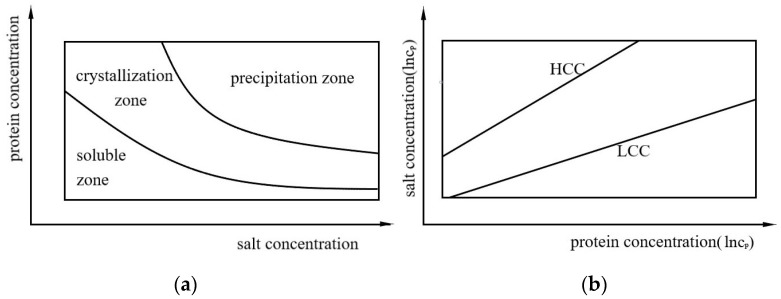
(**a**) A simple model of a phase diagram. (**b**) The reentrant condensation (RC) of a protein.

Proteins dissolve in the undersaturated region and protein crystals grow in the supersaturated region. At equilibrium, the concentration of protein in the solution is dissolved [10,11]. The protein in the precipitation zone comes out of the solution so quickly that there is no time to form a crystal solid [10,12], as shown in Figure 1a. Figure 1b shows RC phase behavior. The plane is divided into three regions by two critical salt concentrations: LLPS will occur between high critical salt concentration (HCC) and low critical salt concentration (LCC).

Protein crystals can form in the protein-rich phase of LLPS, in which the concentration of protein exceeds its solubility limit. It has been shown that the free energy barrier of crystal nucleation decreases significantly at the critical point of liquid–liquid phase separation [13]. Protein phase diagrams containing crystallization and liquid–liquid separation is especially important for the design of protein purification and separation processes by precipitation. Thus, the determination of the location of liquid–liquid phase separation regions is very helpful for identifying the optimal solution conditions for growing protein crystals [14,15,16]. The parameters affecting LLPS of proteins include protein concentration, pH, temperature, salt concentration, additive concentration, and more. It is not easy to predict the solubility directly from these parameters.

In the present work, we use a neural network to construct the hyperplane of the solubility and cloud point temperature of lysozyme—to obtain the phase diagram of lysozyme—and use experimental data to train the network to predict the occurrence of phase separation of bovine serum albumin (BSA) under different conditions. Lysozyme has a variety of antiviral, antitumoral, and immunomodulatory activities and is often used as a model protein in the study of enzyme reactions, protein aggregation, and crystallization [17]. BSA is a globular protein that plays a key role in maintaining plasma pressure and nutritional balance. Theoretically, cloud point temperature can be related to protein concentration via a fitting expression including some parameters. However, these parameters are usually not independent and are unavailable directly. In recent years, artificial neural networks have been widely used in biophysics, such as in the liquid–liquid phase separation of proteins and in polymer simulations [18,19]. The solubility of lysozyme in the NaCl–water system has also been studied by networks [20]. Deep learning has been successfully used as a tool of machine learning, in which neural networks can learn features automatically [21]. Although great progress has been made in this technology for protein crystallization, the successful crystallization of proteins depends to a large extent on experience and operators. The phase diagram is of great help to solving the crystallization problem, and accurate prediction of protein precipitation and crystallization conditions is very important to achieving the goal [22].

## 2. Materials and Methods

### 2.1. Description of Neural Networks

Neural networks, also known as artificial neural networks (ANN), are a computing system that can learn to perform different tasks by considering examples, usually without using task-specific rules for programming. The main advantage of the artificial neural network model is that it has the ability of self-learning and can approach the nonlinear relationship between input variables and output variables of complex systems. The nonlinear activation function in the hidden layer makes the neural network become a general approximator so that it can approach almost any function. In order to ensure a high accuracy of the neural network model, sufficient data set is needed to train the neural network [23].

**Figure 2:**
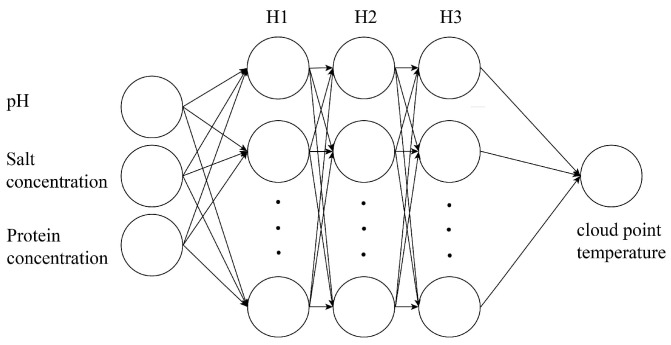
The recurrent neural network model with three hidden layers.

The hierarchical arrangement of a neural network can be divided into an input layer, multiple hidden layers, and an output layer, as shown in Figure 2. There may be many neurons on each layer. Any neuron in the hidden layer and the output layer is connected to all the neurons in the upper layer. Classification and regression are two basic neural network models that utilize different output layers and loss functions. For the lysozyme system, we need to predict its specific cloud point temperature and solubility, so we use the regression model, while the classification model is more suitable for the LLPS prediction of BSA.

All our models have three layers, and the number of hidden units in each layer is different. We trained them with 1000 and 500 epochs on regression and classification, respectively, and evaluated them on the test set. In order to determine the energy metric, we use the mean square error to measure how much the dynamics of a given model deviate from the actual situation. The loss indicator measures the ability of our model to fit a single data point. The loss functions we use in regression are mean square error (MSE) and mean absolute error (MAE). Where Ci is the predicted value and $\hat{\mathrm{Ci}}\;$is the true value. The loss function we use in classification is sparse_categorical_crossentropy.

(1) $$\mathrm{MSE}=\frac{1}{n}\sum_{i}{\left(\mathrm{Ci}-\hat{\mathrm{Ci}}\right)}^{2}$$

(2) $$\mathrm{MAE}=\frac{1}{n}\overset{n}{\underset{i=1}{\sum }}\left|\mathrm{Ci}-\hat{\mathrm{Ci}}\right|$$

The activation functions we use in this work include the sigmoid function, relu function, and softmax function. In addition, we use an adaptive learning rate optimizer, Adam, in training [24]. Hyperparameters, such as the number of neurons, layers, optimizer, and learning rate, are important in training. By using TensorFlow, we focus on the influence of the activation function and the number of neurons in each hidden layer.

### 2.2. Analysis of Data

**Figure 3:**
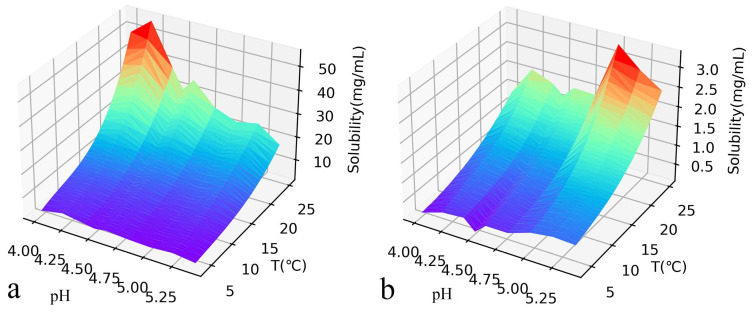
In a 0.1M acetic acid buffer, the concentration of NaCl 2% (**a**) and 7% (**b**), and the effect of temperature and pH value on the solubility of lysozyme. The solubility increased with the increase of temperature and decreased with the increase of salt concentration. The data source is Ref. [11].

Lysozyme is a commonly used protein in many crystallization studies; it is crystallized in tetragonal form. In the crystallization experiments, the solubility of lysozyme was measured at different pHs (4.0–5.4) in a 0.1 M acetic acid buffer [11,25]. It was found that the solubility increases with temperature but decreases with salt concentration. On the other hand, the effect of pH depends on the salt concentration. At low salt concentrations, the solubility goes down with increasing pH, while it goes up at high salt concentrations. The solubility of lysozyme can be approximately fitted by the following expression:

(3) $$C^{\mathrm{solubility}}=A+\mathrm{BT}+{\mathrm{CT}}^{2}+{\mathrm{DT}}^{3}$$

where T is the temperature and A, B, C, and D are coefficients that depend on pH and the concentration of sodium chloride in solution. The Pusey group listed the values of all A, B, C, and D under conditions of pH from 4.0 to 5.4 and temperatures from 4 °C to 24 °C, and we used the same dataset for our network training [11]. However, a set of universal parameters for the solubility of lysozyme for various cases is not yet available. In the present work, we use a neural network to find out this general relationship which can be used even in the unavailable region in experiment. We show the current available solubility data in Figure 3, which corresponds to various temperatures T, sodium chloride concentrations C_NaCl_, and pH values.

The establishment of a database is very important for the training and testing of neural networks. By consulting open literature, we have created a dataset that contains the results of many researchers [26,27,28,29,30,31]. There is no direct answer to the comprehensive functional relationship between lysozyme concentration, pH value, salt concentration C_NaCl_, and cloud point temperature. The current available data were obtained in pH conditions ranging from 4 to 4.8, salt concentrations ranging from 2% to 7% (*w*/*v*), and lysozyme concentrations between 0 and 400 (mg/mL).

At the critical point of liquid–liquid phase separation, characterized by cloud point temperature, the free energy barrier of crystal nucleation decreases significantly, so the crystallization after the point occurs faster than that in the initial solution. The determination of the position of the liquid–liquid phase separation curve and the relationship between protein solubility in specific solvents and temperature is the key to effectively determining the best solution conditions for the growth of protein crystals. Martin Muschol and Franz Rosenberger [26] studied the liquid–liquid phase separation of lysozyme at pH 4.5, measured the cloud point temperature, and gave the fitting function between the cloud point temperature and the lysozyme concentration.

(4) $$T^{\mathrm{cloud}\;}=T^{\mathrm{crit}\;}\left\{1-N{\left|\frac{C^{\mathrm{crit}\;}-C_{\rho }}{C^{\mathrm{crit}\;}}\right|}^{\frac{1}{\beta }}\right\}$$

β = 0.325, and $T^{\mathrm{crit}}\;$(critical temperature) and $C^{\mathrm{crit}}$ (critical concentration) are the cloud point temperature and protein concentration at the critical point, respectively.$\;T^{\mathrm{crit}}$, $C^{\mathrm{crit}}$, and N are related to pH and salt concentration. Nathaniel Wentzel and James D Gunton [30] used the square potential well model to study the relationship between the cloud point temperature and the ionic strength in sodium chloride and magnesium chloride solutions and simulated the liquid–liquid coexistence surface. In their work, the cloud point temperature of the water–sodium chloride system is proportional to the logarithm of the ionic strength.

(5) $$T^{\mathrm{cloud}}\left(I,\rho \right)=\frac{T\left(\rho \right)}{T_{0}}T\left(I\right)$$

(6) $$T\left(I\right)=K\ln I+b$$

I is the ionic strength (M), ρ is the volume fraction of lysozyme, K, b, and T_0_ are parameters related to the experimental conditions. Their study determined that the cloud point temperature is related to both salt concentration and protein concentration, and this model is of great value for the prediction of the cloud point temperature. By determining the parameters, they used Equations (5) and (6) to establish an ideal liquid–liquid coexistence surface with pHs of 4 and 7. These fitting formulas can be applied to most areas by adjusting the parameters.

However, the relationship between cloud point temperature and pH has not been determined yet. Victor G Taratuta [32] studied the effect of sodium phosphate on the cloud point temperature of lysozyme and concluded that $T^{\mathrm{cloud}}$ increases linearly from pH 5.8 to pH 8.0 when the total ionic strength of the buffer remains constant. However, this conclusion is not completely applicable at low pH. Through the experimental data available and using the above formula to fit some missing points, we build a relatively complete data set, including 1600 training samples of solubility data and 1000 training samples of cloud point temperature used to train the neural network.

The LLPS data of BSA were obtained from the experiments of our research group [33]. When DDAO was added to the mixed solution of BSA–YCl_3_, the low critical salt concentration (LCC) for liquid–liquid phase separation of BSA is almost unchanged, but the high critical salt concentration (HCC) is dependent on the concentration of DDAO. Low concentration of DDAO reduces HCC and thus inhibits LLPS, while high concentration of DDAO increases HCC and promotes LLPS. The experimental temperature is set to 40 °C. The data have a total of 2000 groups, covering the range of low concentrations of DDAO (0–10 mM), YCl_3_ (3–20 mM), and BSA (30–100 mg/mL) and high concentrations of DDAO (1–60 mM), YCl_3_ (1–12 mM), and BSA (40mg/mL).

### 2.3. Experimental Materials

In order to check the accuracy of our network prediction, we performed the liquid–liquid phase separation of lysozyme and BSA in the lab. The source of experimental materials is listed as follows: chicken egg-white lysozyme was purchased from Sigma Chemicals (lot SLCC4285) and BSA was purchased from Sigma Chemicals (lot WXBD0807V) (St. Louis, MO, USA). Sodium acetate was purchased from Sangon Chemicals (lot 1437B508)(Shanghai, China). Acetic acid was purchased from Sinopharm Chemicals (lot T20080203) (Beijing, China). NaCl was purchased from Sigma Chemicals (lot SZBD2390V). YCl_3_ was purchased from Sigma Chemicals (lot 26096). DDAO were purchased from Sigma at high-purity grade (>99%). Purified water was obtained from the Milli-Q system (Millipore, Billerica, MA, USA). We used a Nikon Ti-E inverted microscope to observe the phase separation of lysozyme and BSA. This microscope is equipped with a Nikon 100× oil-immersion objective (Nikon CFI Apo 100XW NIR, Tokyo, Japan), a 48MP FHD Camera V8, and a Instec MK2000B temperature controller.

## 3. Results and Discussion

### 3.1. Prediction by ANNs

By training the neural network described in Section 2.1, we can predict the phase behavior of lysozyme and BSA. For the prediction of solubility and CPT, we chose the ADAM optimizer, with a fixed learning rate of 10^−1^, 3 hidden layers, and 64 neurons per layer. In order to solve the classification of BSA, the three hidden layers of our neural network have 128, 64, and 32 neurons respectively, the output layer has 3 neurons and uses softmax as the activation function, thus outputting the probability that LLPS occurs and LLPS does not occur when it is higher than HCC or lower than LCC. We used dropout to prevent overfitting in the neural network, which was further improved by adjusting and using appropriate regularization parameters to limit the weights.

**Figure 4:**
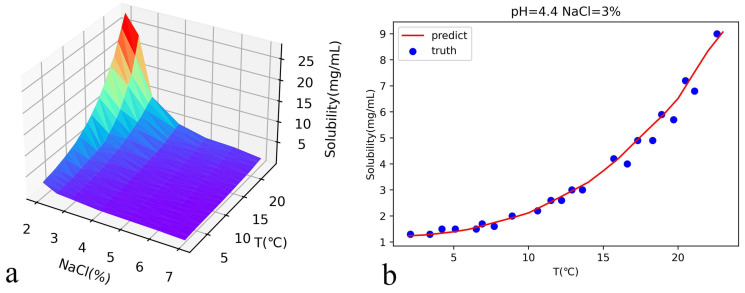
(**a**) pH = 4.4, the solubility of lysozyme is predicted by the neural network. (**b**) shows the comparison between the predicted results and the experimental values in Ref. [25] when the concentration of sodium chloride is 3%. The MAE between the predicted and experimental values is 0.2.

The predicted solubility of lysozyme at pH = 4.4 is shown in Figure 4a. In order to check the prediction accuracy, we show the comparison between the prediction curve with the experimental values [25]. We can see that the neural network works well although the experimental values fluctuate around the prediction curve slightly.

**Figure 5:**
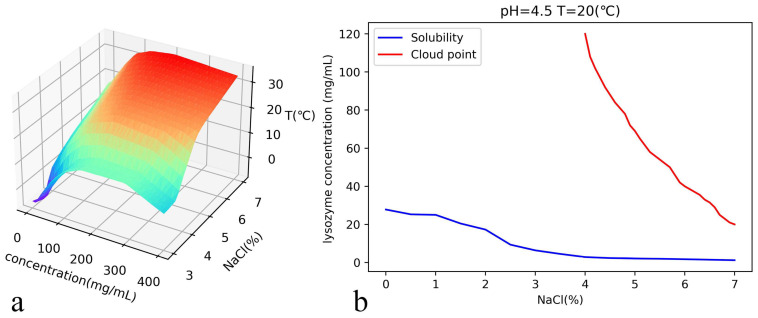
(**a**) The cloud point temperature map of the whole space when pH = 4.5 is established by using the neural network. (**b**) The phase diagram is made by using the neural network when the the pH is 4.5 and the temperature is 20 °C. The neural network can be used to draw the phase diagram under given conditions in a certain range.

**Figure 6:**
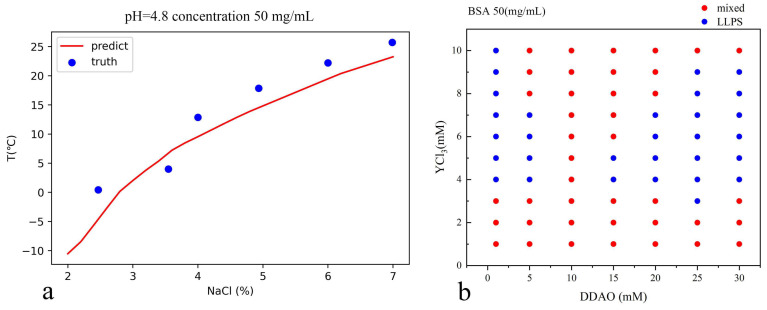
(**a**) pH 4.8 and lysozyme concentration 50 mg/mL, the cloud point temperature is compared with the experimental data from Ref. [28]. The MAE between the predicted and experimental values is 3.2. (**b**) BSA 50 mg/mL, the predicted results at different concentrations.

Figure 5a shows the predicted cloud temperatures in a wide range of lysozyme and salt concentrations, while Figure 5b shows the solubility and cloud point curves predicted by the trained network. The training was carried out through limited specific experimental data since not so many are available. Even so, the predicted values can still provide valuable information in a wide range of concentrations. Specifically, we provide the comparison between the predicted and experimental data at pH 4.8 in Figure 6a. We can see that the predicted value of the neural network does not completely match the experimental data, but it is still quite reliable. We must point out that this part of the experimental data does not participate in the data training.

Figure 6b shows the prediction results of LLPS of BSA by neural network. These prediction results show two basic characteristics of the training set data: DDAO inhibits phase separation at low concentrations (0–10 mM) and promotes phase separation at high concentrations (10–30 mM). With the increase of salt concentration, the solution will change from mixed state to LLPS and then return to a mixed state. There is no phase separation data of BSA 50 mg/mL at high concentrations of DDAO in the training set, so we will verify the prediction results through experiments.

### 3.2. Accuracy and Loss Functions

**Figure 7:**
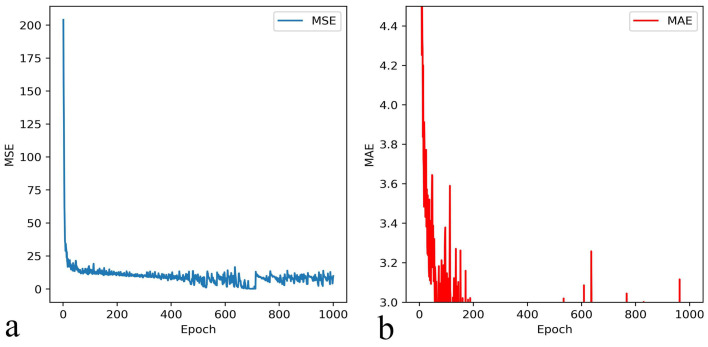
The loss functions MSE (**a**) and MAE (**b**) converge with the increase of epoch.

The loss function can directly reflect the training results of the neural network, which is helpful to adjust the hyperparameter and help the model converge faster. We used L1 regularization twice, and the regularization parameter was set to 0.1. After 1000 epochs of training, we reduced the loss function MSE to 10^−1^. MSE and MAE in the training are shown in Figure 7.

**Figure 8:**
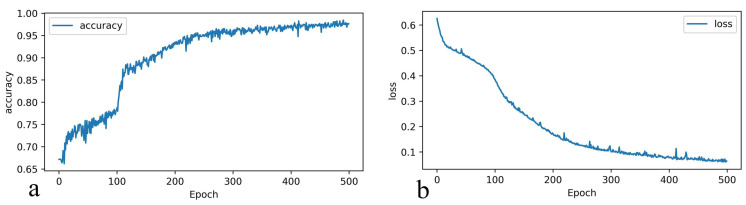
The results of classification of BSA phase separation processed by the neural network. The accuracy and loss functions sparse_categorical_crossentropy converge with the increase of epoch. With the increase of the epoch of training, the accuracy increases (**a**) and the loss function decreases (**b**).

Accuracy is the most intuitive criterion for judging classification problems. We show the accuracy and loss function in the training in Figure 8. After the training, the prediction accuracy for LLPS of BSA is 0.98 for the training set and 0.94 for the testing set. After 500 epochs of training, the loss function is reduced to 0.062.

### 3.3. Experimental Verification

**Figure 9:**
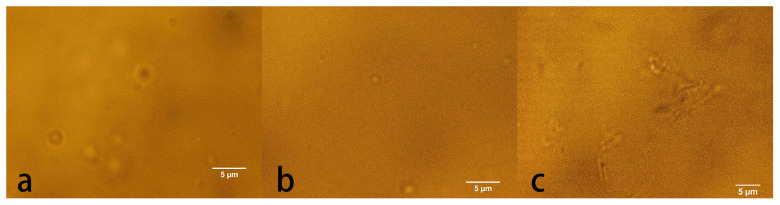
Phase separation of lysozyme under microscope at 25 °C pH 4.8. (**a**) lysozyme concentration 120 mg/mL, NaCl 4%; (**b**) lysozyme concentration 60 mg/mL, NaCl 2%; (**c**) lysozyme concentration 20 mg/mL, NaCl 8%.

In order to verify the prediction of phase behavior of lysozyme and BSA, we conducted quite a few experiments at these predicting points. Figure 9 shows the results of microscopic observation at different concentrations of salt and lysozyme. We can see that liquid–liquid phase separation occurs in (a) and (b), while precipitation can be found in (c), which are all consistent with the predictions of our neural network.

**Figure 10:**
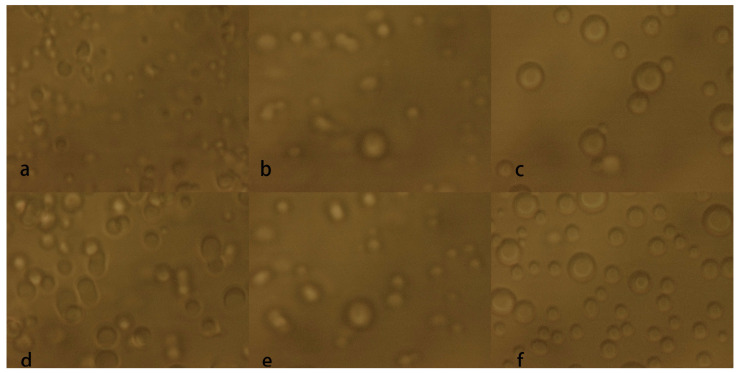
The LLPS comparison diagram of BSA under the condition of a fixed BSA concentration 50 mg/mL. (**a**) YCl_3_ 3 mM, DDAO 3mM; (**b**) YCl_3_ 3 mM, DDAO 30 mM; (**c**) YCl_3_ 3 mM, DDAO 50 mM; (**d**) YCl_3_ 7 mM, DDAO 3 mM; (**e**) YCl_3_ 7 mM, DDAO 30 mM; (**f**) YCl_3_ 7 mM, DDAO 50 mM.

For the BSA system, our group recently showed that low concentrations of DDAO inhibit phase separation and high concentrations promote phase separation. Using this neural network, we predict the phase separation of BSA at a concentration of 50 mg/mL with different concentrations of DDAO, and tested the prediction by experiments directly. As shown in Figure 10a,b, and c are the results of microscopic observation when YCl_3_ is 3 mM and the concentration of DDAO is at 3 mM, 30 mM, and 50 mM, respectively. Figure 10d,e, and f are the results of microscopic observation when YCl_3_ is 7 mM and the concentration of DDAO is 3 mM, 30 mM, and 50 mM, respectively. Obvious phase separation can be seen in these images, which are fully consistent with the prediction. It is notable that the current 50 mM BSA concentration is not included in our previous experiments.

## 4. Conclusions

In summary, we trained fully connected regression and classification of neural networks to predict the liquid–liquid phase separation of lysozyme and bovine serum albumin (BSA) both qualitatively and quantitively. Specifically, we predicted the cloud point and solubility of lysozyme solution containing sodium chloride and the upper and lower limits of protein concentration for LLPS. In addition, we also predicted the reentrant phase behavior of BSA in YCl_3_ solution containing a surfactant dodecyl dimethyl amine oxide (DDAO). Our network works well in a broad range of pHs (4.0–5.4), temperatures (0–25 °C) and NaCl concentrations (2–7% *w*/*v*) for the lysozyme system, while in ranges of DDAO (1–60 mM), BSA (30–100 mg/mL), and YCl_3_ (1–20 mM) for the reentrant phase transition of BSA. We tested the model experimentally and found that the predicted results of the neural network are highly consistent with the previous numerical analysis methods and current experimental results.
